# Food Adulteration in 1902

**Published:** 1903-11-28

**Authors:** 


### The Hospital.

A Journal of

The Medical Sciences and Hospital Administration.

Vol. XXXV.—No. 896. NOVEMBER 28, 1903.

### Food Adulteration in 1902.

The report for 1902 of the work of the Local Government Board, full as it is of interesting matter on a great variety of subjects, deals with few that are of greater importance to the community than the administration of the Food and Drugs Act, under which 231 regularly-appointed analysts are now discharging their duties in the City of London, in the metropolitan boroughs, and in administrative counties, county boroughs, and non-county boroughs in different parts of the kingdom. The total number of samples analysed in 1902 amounted to 72,321, or to one sample for every 450 of the population of the last census. In London, where the inspectors are more active than elsewhere, the analyses were at the rate of 4 per 1,000 of the population; and in the provinces they were at the rate of about 2 per 1,000. The Board has communicated from time to time with the local authorities of districts in which the number of samples submitted for examination was unduly low, and has generally succeeded in stimulating them to increased activity. In 1898 there were 93 districts in which the number of samples analysed did not amount' to 1 per 1,000 of population. In 1902 the number of these districts was reduced to 25; and, for the first time since the passing of the Act of 1875, there was not a single district from which no analysis was reported. Of the 72,321 samples examined in 1902 adulteration was discovered in 6,281, and legal proceedings were instituted in respect of 3,556. Penalties were imposed in 2,928 cases, amounting in the aggregate to £7,064 5s. 4d., excluding costs in most instances; but these penalties were in many cases too small to be deterrent. During the year the Board drew the attention of the Home Secretary to the complaints made by local authorities of the conduct of many Justices in this respect, and he consequently addressed a circular letter to clerks of petty sessional divisions throughout the country, asking that the matter might be brought to the notice of the Justices, and that they would give special consideration to the question whether the penalties for adulteration ordinarily imposed by them were such as would be likely to deter the convicted persons from repeating, or other persons from committing, the offences for which these penalties were inflicted. The need for such a circular is well illustrated by the statement of the public analyst for Walsall, who reports that he condemned a sample of milk because it contained 7 per cent. of added water, and that the magistrate before whom the consequent proceedings were taken dismissed the case without hearing the defence, and remarked that the prosecution was a paltry one.

The whole case of milk adulteration appears from the report to be a very serious one, both as regards frequency and character, and also, as need hardly be said, in view of the enormous importance of the commodity as an element in the food of children. Nearly five-twelfths of all the samples taken in 1902 were of milk, and of these 11·6 per cent. were reported against. This is the highest rate of milk adulteration recorded in any year since 1893, and it appears to be due to a systematic endeavour to reduce the quality of good milk so that it may just reach the standard fixed by the Board of Agriculture. This is done either by the addition of water, the addition of separated milk, or the removal of cream; insomuch that the old and gross form of adulteration by the rule of thumb addition of water is largely giving place to more refined, and, so to speak, scientifically adjusted methods, by the employment of which the producer, with absolute impunity, gets the full extra profit of all the cream his milk naturally contains over and above the minimum yielded by a poverty-stricken cow, and accepted by the Board of Agriculture as a standard. In this matter London appears to be leading the way; its percentage of adulteration being 15·6 as against 10·3 in the 20 next largest towns of England and Wales, and 10·0 in the rest of the country. In six of the metropolitan boroughs the proportion of adulterated samples exceeded 20 per cent. Out of 176 samples of condensed milk 17 were condemned, and 69 samples of cream out of 150 were reported to contain boric acid. It is more pleasant to record that there were 1,545 convictions for milk adulteration, and that the penalties, which in 266 cases were of £5 or upwards, amounted in the aggregate to £3,783 5s. 6d. It may be feared, nevertheless, that the penalties bear a very small proportion to the profits of these nefarious practices; and it does not seem likely that the public will be effectually protected unless or until the retailers of milk are required to take out an excise license, which would be liable to forfeiture on conviction. The adulteration next in importance appears to be that of margarine, effected by the addition of water. Water, it must be remembered, is not intrinsic to margarine, and every drop of it has been added for the sole purpose of being sold by weight at the price of margarine. In four different samples water was found to the extent respectively of 16 1, 18·11, 20·82, and 21·44 per cent.; and adulteration was found in 81 samples out of 1,048 examined, or in nearly 8 per cent. Glancing at the returns with regard to other articles, we find that adulteration was discovered in 6·5 per cent. of the samples of butter, 7·3 per cent. of coffee, 13 3 of cocoa, 8·2 of sugar, 5·1 of mustard, 2·8 of confectionery and jam, 26·5 of wine, 3·5 of beer, and 12·3 of spirits. Among drugs, adulteration was found in 23·7 per cent. of the samples of "nitre," a word not more fully explained; in 11·1 per cent. of the seidlitz powders, in 17·1 per cent. of magnesia, and in 25·8 per cent. of mercurial ointment, a preparation largely purchased by certain classes of the town poor as an insecticide. These things, however, are comparatively unimportant when compared with staple articles of the food of the population.

